# Experiences of violence among people with stimulant use disorder in psychiatric inpatient settings: A qualitative study

**DOI:** 10.1177/10398562231196672

**Published:** 2023-08-22

**Authors:** Matthew Tennant, Marie Crowe, James Foulds

**Affiliations:** Department of Psychological Medicine, 2495 University of Otago, Christchurch, New Zealand

**Keywords:** Stimulant use disorder, violence, trauma-informed care, qualitative research, methamphetamine

## Abstract

**Objective:**

To describe the perspectives of those with lived experience of stimulant use disorder on methamphetamine-related violence in psychiatric inpatient settings.

**Method:**

Eight adult psychiatric inpatients with stimulant use disorder were recruited. Semi-structured interviews were recorded, transcribed and analysed using thematic analysis.

**Results:**

Participants reported that traumatic experiences predisposed those using methamphetamine to violent behaviour. Participants were fearful of psychiatric hospitalisation because of loss of autonomy and stigma. Methamphetamine use was associated with mercurial intense emotions. Participants believed these factors led to violence during psychiatric admissions.

**Conclusions:**

People with stimulant use disorder have a sophisticated understanding of the complex causal pathways from methamphetamine use to violent behaviour. Their lived experience can make an important contribution to service development.

Methamphetamine-related psychiatric admissions have increased steeply in recent years as high-potency methamphetamine has become cheaper and more available.^1^ A recent Australian study published in Australasian Psychiatry reported that 21.7% of acute psychiatric admissions were related to methamphetamine use.^2^

The expanding number of people with stimulant use disorder (SUD) accessing acute health services has created challenges, including how best to manage violence. While the perspectives of clinicians have been studied,^3^ few studies have considered the experience of people with SUD when they access acute mental health care. A better understanding of this lived experience would help inform treatment approaches that enhance consumers’ long-term recovery.^4^

## Methods

### Ethical and cultural consideration

This study followed the New Zealand Health and Disability Ethics Committee (HDEC) minimal risk observational study pathway with organisational approval provided (RO20040). Written informed consent was given by participants. Consultation with Maori health advisers ensured the study’s methods were congruent with obligations under Te Tiriti o Waitangi.

### Qualitative approach

Our process was underpinned by a critical realist theoretical position and an ontological position that assumes that people’s words provide access to their particular version of reality.

### Researcher characteristics

The primary researcher (Author 1) was an immigrant male psychiatric registrar of European heritage in his 30s. Secondary authors included a professor of psychiatric nursing and a consultant psychiatrist.

### Setting and Sampling

Participants were recruited via convenience sampling from General Adult and Forensic Mental Health Services in Christchurch, New Zealand. The study aimed to recruit eight to twelve participants with a history of an inpatient admission in the context of stimulant use and a lifetime history of SUD. The diagnosis was confirmed using DSM-5 criteria prior to the qualitative interview.^5^ Due to challenges with recruitment and early analysis suggesting data saturation, we stopped recruiting at eight participants. Purposive sampling of four participants identifying as Māori incorporated an indigenous perspective, as Maori are disproportionally affected by SUD.^6^ Participants did not receive any financial compensation for their involvement. No participants were under the clinical care of any of the authors at the time of the study but two were previous patients of one author (JF).

### Data collection

Individual semi-structured interviews were designed to elicit in-depth descriptions of the participants’ experiences of methamphetamine use, mental health inpatient admissions, and violence related to either methamphetamine use or inpatient admissions. Participants were asked first about their own experiences of behaving violently, and then if this line of questioning was poorly tolerated, they were asked to discuss violence observed. Interviews were conducted by the primary researcher (Author 1) and were between 35 and 70 min duration. They were held in a private interview room between May and November 2020. Interviews were audio recorded and then transcribed in a de-identified form.

### Data analysis

Thematic analysis as described by Braun and Clarke^7^ was applied. Data were coded manually by one author (MT) and then re-examined for any missed or latent content. Coded data were organised using Microsoft Excel. A thematic map was used to visualise the data as a whole and then form thematic clusters of related ideas. Another author (MC) reviewed the data, and initial themes were defined and named through discussion. Themes were refined through a review of the relevant transcript data and illustrative quotes were selected.

## Findings

The sample was composed of six males and two females ranging from their early twenties to late fifties (see Table 1). Four were diagnosed with substance-induced psychotic disorder. The others had primary diagnoses of schizophrenia, schizoaffective disorder, bipolar I disorder and major depressive disorder. Methamphetamine was the primary stimulant used by all participants.

**Table 1.** Sample demographics.

	Participants = 8
Gender	
Female	2
Male	6
Age range	
18–39	4
39–60	4
Ethnicity	
Maori	4
NZ European	6
Australian	1
Primary diagnosis	
Brief psychotic episode (drug induced)	4
Schizophrenia	1
Schizoaffective disorder	1
Bipolar I disorder	1
Major depressive disorder	1
Legal status (most recent psychiatric admission)	
Mental Health Act	5
Voluntary/informal	3
Hospital unit	
General adult	6
Forensic adult	2
Seclusion (utilised within duration of most recent admission)	
Yes	2
No	6

Three core themes were identified from nineteen codes and seven sub-themes (see Table 2). They were: ‘Every “addict” [sic] has a trauma’, ‘Scared of a hospital sentence’ and ‘That’s why he raged’.

**Table 2.** Theme development.

Codes	Sub-themes	Theme
Childhood trauma Exposure to criminality Expecting violence ‘Angry people get angry’	Trauma Anger	‘Every addict has a trauma’
Anxiety about stigma Fear of compulsory treatment Invasion of privacy Disempowerment Needing space	Stigma Coercion	‘Scared of a hospital sentence’
Mercurial affects Grandiosity/invincibility Comedown irritability/dysphoria Sleep deprivation Hunger Anxiety and panic Dose response Mixing substances	Impulsive Strong emotions Dose and mixing substances	‘That’s why he’d rage’

### ‘Every “addict” [sic] has a trauma’

This theme reflects participants’ descriptions of past trauma and the stigma of being a person with an addiction. Most participants described violence as associated with past trauma which had an on-going impact on their lives. They reported that staff often blamed violence on drug intoxication rather than acknowledging the contribution of adverse life experiences.

‘I would be like see through the window, them kicking the shit out of their wife because they are having a bad come down. Then they would blame the drug. But it wasn’t the drug that did it. They were already damaged and messed up and violent’ (Participant 7).

Participants suggested substance intoxication helped people deal with previous trauma. It was apparent many thought the history of trauma was more related to violence than methamphetamine.

‘Basically, every addict will have some sort of trauma that has led them to be at a point where they use the substance in a way that affects their lives inappropriately, they can’t stop… so, I think that people think that substance is the cause, not why did you need that in the first place’ (Participant 8).

### ‘Scared of a hospital sentence’

Participants feared psychiatric hospitalisation. They were unsure what to expect on the ward and feared the effects of sedative medications that might be given under compulsion.

‘I was actually really scared of a hospital sentence, didn’t know what to expect, scared they were going to give me like a chemical lobotomy, put me on drugs that’s gonna mess with my mind so I won’t be myself, I was very afraid of the system, the mental health system’ (Participant 7).

Psychiatric assessment was perceived as invasive. Participants often felt staff and family were colluding to have them admitted.

‘I was coaxed here by my mum and others…I felt a wee bit trapped, even though I did volunteer myself’ (Participant 5).

Inpatient wards had rules and expectations regarding when and how activities were done, which participants experienced as disempowering.

‘Just the fear of not being in control of your own life. All of a sudden, you’ve got these controls or a whole new set of rules’ (Participant 7).

### ‘That’s why he’d rage’

Participants described mercurial affects during intoxication and withdrawal. They reported dysphoria and irritability when first admitted to a psychiatric ward due to methamphetamine withdrawal.

‘Yeah, I think the come down you’re far more dangerous. When life just sort of all falls out from underneath you, you become a walking hazard’ (Participant 6).

Withdrawal was exacerbated by sleep deprivation and hunger. They suggested that prioritising sleep and food early in admission could reduce violence.

‘So imagine, what would you feel like after 12 hours of not eating, fasting? You know what I mean? So you actually go into a low mood anyway and then you got that come down on top of it…’ (Participant 6).

Some observed that when using methamphetamine, they did not notice the effects of alcohol intoxication. As a result, some reported consuming more alcohol when they were using methamphetamine. Participants associated increased alcohol with behavioural disinhibition and violence.

‘He drunk, he used, that’s why he’d rage’. (Participant 1).

Participants identified intense unstable emotional states while using and during withdrawal from methamphetamine. This could increase the risk of violent behaviour during the period of withdrawal often experienced on psychiatric units.

## Discussion

Participants had a sophisticated understanding of the impact of psychiatric hospitalisation on people with SUD. They provided ideas on simple early interventions that would help adjustment to hospital. They emphasised trauma-informed care principles including the importance of staff attending early to basic needs for food, sleep and a quiet, non-threatening environment. Participants had explanatory models that recognised the role of distal factors including adverse life experiences, personality and environmental factors in shaping violent behaviour. This nuanced view contrasts with the common perception among health staff and the general public that violence associated with stimulant use is mainly driven by intoxication.^3^

Our findings were consistent with two previous community studies. Californian youth who had committed violent acts described a pattern of violent behaviours preceding methamphetamine use, suggesting developmental factors contribute to violence in people using methamphetamine.^8^ In a second study, arrestees who were using methamphetamine believed that hostile environments encountered while accessing methamphetamine precipitated violence.^9^

The strength of this study is that it provides an authentic voice for a rapidly growing group who are under-represented in the existing literature. The results support the movement to trauma-informed care. Clinicians need to be aware that antecedent individual factors, the health care environment and the way staff respond to individual patients may shape violent behaviour among people with SUD on inpatient units. A better understanding of these complex, dynamic processes would help improve the care. A collaborative approach that includes the voice of service users would help improve the quality of care and create safer inpatient environments.

Strategies to improve care for this group include focusing on meeting patients’ basic biological needs early in inpatient admissions; building trust by providing clear explanations and transparency about treatment decisions; empowering staff to support cultural change within the workplace and increasing training and support for health staff.^10^

This was a small qualitative study and caution is needed in generalising these exploratory findings to other settings. A further limitation was that interviews focused on participants’ beliefs about violence, but they did not dissect the context of actual violent incidents within inpatient settings. However, while most participants found it difficult to talk about their own violent actions, they were willing to discuss violence they had witnessed and to share their own explanatory models. Participants were not asked to review the transcripts and themes. Greater participant involvement at these stages might have improved the study’s validity.

## Conclusion

People with SUD have a sophisticated understanding of the complex causal pathways from methamphetamine use to violent behaviour that is not well captured in quantitative research. The lived experience and knowledge of people with SUD can make an important contribution to service development for the rapidly growing group of people with SUD coming in contact with acute mental health services.
